# Supplementary material for: Repeated Antigen-Based Rapid Diagnostic Testing for Estimating the Coronavirus Disease 2019 Prevalence from the Perspective of the Workers’ Vulnerability before and during the Lockdown
Source: Int J Environ Res Public Health. 2021 Feb 9;18(4):1638. doi: 10.3390/ijerph18041638 (PMC7915907; doi:10.3390/ijerph18041638)
Supplement: Supplementary file 1 [file ijerph-18-01638-s001.pdf]

Supplementary Table S1. Characteristics of the study population stratified by gender, Pre-lockdown screening program (N=1054)

**Study Characteristics**	**Total (n=1054)**	**Males (n=536)**	**Females (n=517)**	**p-value**
Age (years)	42±19	41±20	42±18	0.406
Medication, (yes), n (%)	367 (34.8)	178 (33.2)	189 (36.6)	0.151
Non-smokers, n (%)	606 (57.5)	300 (60.0)	306 (59.2)	0.345
Current smokers, n (%)	326 (30.9)	161 (30.0)	165 (32.0)	0.452
Ex-smokers, n (%)	88 (8.3)	57 (10.6)	31 (6.0)	0.05
Respiratory symptoms, (yes), n (%) *the last 15 days	238 (22.6)	123 (22.9)	115 (22.2)	0.421
Previous contacts with a Covid-19 positive case, n (%)	357 (33.9)	171 (31.9)	186 (36.0)	0.092
Friendly Environment, n (%)	112 (10.6)	62 (11.6)	50 (9.7)	0.185
Family Environment, n (%)	90 (8.5)	35 (6.5)	55 (10.6)	0.011
Professional Environment, n (%)	104 (9.9)	49 (9.1)	55 (10.6)	0.239
Leisure environment, n (%)	57 (5.4)	31 (5.7)	26 (5.0)	0.343
>1 previous contacts, n (%)	8 (0.7)	5 (0.9)	3 (0.05)	0.383
Travels *the last 15 days	141 (13.4)	76 (14.2)	65 (12.6)	0.692
Previous testing, (yes)<br>(average: 2±1 tests, max=6)	195 (18.5)	97 (18.1)	98 (19)	0.390

***Notes:** Data are expressed as mean ± standard deviation or as frequency (percentage).*

Supplementary Table S2 Characteristics of the study population stratified by gender, Second screening program (N=462)

**Study Characteristics**	**Total (n=462)**	**Males (n=240)**	**Females (n=221)**	**p-value**
Age (years)	48±17	49.4±16.4	46.4±17.5	0.05
Medication, (yes), n (%)	144 (31.2)	79 (32.9)	65 (29.4)	0.248
Non-smokers, n (%)	267 (57.9)	136 (56.7)	131 (59.3)	0.300
Current smokers, n (%)	137 (29.7)	67 (27.9)	70 (31.7)	0.209
Ex-smokers, n (%)	58 (12.6)	38 (15.8)	20 (9.0)	0.02
Symptoms, (yes), n (%) *the last 15 days	88 (19.1)	40 (16.6)	48 (21.7)	0.129
Previous contacts with a Covid-19 positive case, n (%)	108 (23.4)	43 (17.9)	65 (29.4)	0.009
Friendly environment, n (%)	24 (5.2)	9 (3.8)	15 (6.8)	0.102
Family environment, n (%)	63 (13.7)	21 (8.8)	42 (10.9)	0.001
Professional environment, n (%)	24 (5.2)	13 (5.4)	11 (5.0)	0.504
Leisure environment, n (%)	13 (2.8)	9 (3.8)	4 (1.8)	0.167
>1 previous contacts, n (%)	9 (2.0)	4 (1.7)	5 (2.3)	0.446
Travels*the last 15 days	14 (3.0)	9 (3.8)	5 (2.3)	0.259
Previous testing, (yes), n (%) (average: 2±1, max=7)	170 (36.9)	94 (39.2)	76 (34.4)	0.176
Previous positive tests, n (%)	31/170 (18.2)	19/94 (20.2)	12/76 (15.8)	0.295
Supermarket visit, (yes), n (%)	318 (68.8)	169 (70.4)	149 (67.4)	0.299
Supermarket visits (frequency)	3±2	4±3	3±2	0.001

Notes: *Data are expressed as mean ± standard deviation or as frequency (percentage).*
